# Potential Role of Probiotics in Ameliorating Psoriasis by Modulating Gut Microbiota in Imiquimod-Induced Psoriasis-Like Mice

**DOI:** 10.3390/nu13062010

**Published:** 2021-06-11

**Authors:** Wenwei Lu, Yadan Deng, Zhifeng Fang, Qixiao Zhai, Shumao Cui, Jianxin Zhao, Wei Chen, Hao Zhang

**Affiliations:** 1 State Key Laboratory of Food Science and Technology, Jiangnan University, Wuxi 214122, China; luwenwei@jiangnan.edu.cn (W.L.); yadand@foxmail.com (Y.D.); zhifengf@foxmail.com (Z.F.); zhaiqixiao@jiangnan.edu.cn (Q.Z.); cuishumao@jiangnan.edu.cn (S.C.); zhaojianxin@jiangnan.edu.cn (J.Z.); chenwei66@jiangnan.edu.cn (W.C.); 2 School of Food Science and Technology, Jiangnan University, Wuxi 214122, China; 3 National Engineering Research Center for Functional Food, Jiangnan University, Wuxi 214122, China

**Keywords:** psoriasis, probiotics, IL-23/Th17 axis, gut microbiota

## Abstract

Psoriasis is an immune-mediated systemic disease that may be treated with probiotics. In this study, probiotic strains that could or could not decrease interleukin (IL)-17 levels were applied to imiquimod (IMQ)-induced psoriasis-like mice via oral administration. *Bifidobacterium adolescentis* CCFM667, *B. breve* CCFM1078, *Lacticaseibacillus paracasei* CCFM1074, and *Limosilactobacillus reuteri* CCFM1132 ameliorated psoriasis-like pathological characteristics and suppressed the release of IL-23/T helper cell 17 (Th17) axis-related inflammatory cytokines, whereas *B. animalis* CCFM1148, *L. paracasei* CCFM1147, and *L. reuteri* CCFM1040 neither alleviated the pathological characteristics nor reduced the levels of inflammatory cytokines. All effective strains increased the contents of short-chain fatty acids, which were negatively correlated with the levels of inflammatory cytokines. By performing 16S rRNA gene sequencing, the diversity of gut microbiota in psoriasis-like mice was found to decrease, but all effective strains made some specific changes to the composition of gut microbiota compared to the ineffective strains. Furthermore, except for *B. breve* CCFM1078, all other effective strains decreased the abundance of the family Rikenellaceae, which was positively correlated with psoriasis-like pathological characteristics and was negatively correlated with propionate levels. These findings demonstrated effects of strain-specificity, and how probiotics ameliorated psoriasis and provide new possibilities for the treatment of psoriasis.

## 1. Introduction

Psoriasis is a skin disease with erythema and scales as the main clinical manifestations. Psoriasis was originally thought to be a disease of epidermal keratinocytes, but is now considered one of the most common immune-mediated diseases [1]. The prevalence of psoriasis worldwide is approximately 2%, but varies according to different regions [2]. Although psoriasis occurs at any age, its incidence is highest between 18 and 39 years of age, or between 50 and 69 years of age [3]. The typical clinical manifestations of psoriasis are scales and erythema, localised or widely distributed [4]. According to the different clinical manifestations of diseased skin, psoriasis is classified as plaque-type psoriasis, guttate psoriasis, inverse psoriasis, pustular psoriasis, palmoplantar psoriasis, and erythrodermic psoriasis [5]. Although there have been a large number of studies on psoriasis, the pathogenesis of psoriasis is not fully understood. It has been recently confirmed that the pathogenesis of psoriasis is not due to a single cause, and involves many aspects, including genetic, immunological, environmental, and other factors [6]. Additionally, psoriasis is an innate and adaptive immune system disease in which keratinocytes, dendritic cells, and T cells play a central role [7]. Several abnormalities occur during the development of psoriasis, including in antigen presentation, activation of the nuclear factor kappa-B signalling pathway, T helper (Th) cells population differentiation (especially Th17 cells), and enhancement of IL-17 responses [6,8]. Recently, more attention has been paid to the interleukin (IL)-23/Th17 axis in psoriasis. IL-23 drives the differentiation of Th17 cells, and Th17 cells subsequently produce IL-22 and IL-17 [9]. These inflammatory cytokines, especially IL-17, accelerate the development of psoriasis [10]. Due to the complexity of psoriasis pathogenesis, there are no methods to completely cure and not relapse after the drug is stopped. Biologics are current the most effective method for treating psoriasis and having certain safety. However, biological preparations are usually expensive. Many kin of biologics, including anti-IL-12/23p40 antibody and IL-17 inhibitors [11], have been used to treat psoriasis. The application of IL-17 inhibitors, including secukinumab, ixekizumab, and brodalumab [11], has confirmed that IL-17 is a therapeutic target for psoriasis. Therefore, we hypothesised that probiotics that suppress the release of IL-17 may be used to treat psoriasis.

The gut is an important digestive organ, and a microbiota niche. The gut microbiota population is in dynamic equilibrium, with many functions, including resistance to pathogen invasion, maintenance of gut homeostasis [12], metabolism of carbon and nitrogen [13], and nourishment of the host [14]; therefore, human health is closely associated with gut microbial alteration. In addition, the gut microbiota regulates immunity through its metabolites; short-chain fatty acids (SCFAs), products of carbohydrate digestion by gut microbiota, promote T cell differentiation into regulatory T (Treg) cells [15], and this in turn blocks the differentiation of other T cells, such as Th17 cells [16]. A large number of studies have reported that there are gut microbial disorders in patients with psoriasis. Hidalgo et al. [17] and Scher et al. [18] have both reported a significant decrease in gut microbial diversity. At the phylum level, the percentage of Firmicutes significantly increases while that of Bacteroidetes significantly decreases, contributing to an elevated ratio of Firmicutes/Bacteroidetes; additionally, there is a positive correlation between the ratio and psoriasis severity [19,20]. At the genus level, the relative abundance of *Akkermansia* and *Ruminococcus* significantly decrease; these genera may produce SCFAs and then induce Treg differentiation or maintain intestinal homeostasis [18,21]. At the species level, the relative abundance of *Akkermansia muciniphila* and *Faecalibacterium prausnitzii* significantly decreased; both are known as beneficial gut microbiota [21,22]. Therefore, changes in gut microbiota are important for psoriasis development, and regulating gut microbiota may be an alternative treatment for psoriasis.

Severe psoriasis has been related to nutrient deficiency, because hyperproliferation and desquamation of the skin epidermis will accelerate nutrient loss [23]. Besides this, reports in the literature have proven that certain dietary patterns have anti-inflammatory effects, and thus have the effect of alleviating psoriasis [24]. Therefore, it is feasible to improve psoriasis by adjusting diet. Probiotics are defined as living microorganisms that have a beneficial effect on the health of the host when ingested in sufficient quantities. The main food source of a probiotic is dairy products. Probiotics also have the effect of improving gut microbial homeostasis and have potential immunomodulatory effects [25,26]. Chen et al. have applied *Lactobacillus pentosus* GMNL-77 to imiquimod (IMQ)-induced psoriasis-like mice through intragastric administration, resulting in reduced pathological features in mice [27]. Rather et al. have applied *L. sakei* proBio-65 extract to IMQ-induced psoriasis-like mice through topical application, which also resulted in reduced pathological features in mice [28]. Although the mechanism was not spelled out, both studies found that levels of IL-23/Th17 axis-related inflammatory cytokines, including IL-23, IL-22, and IL-17, decreased after probiotic treatment. Therefore, we hypothesize that increasing the intake of foods with anti-inflammatory probiotics can ameliorate psoriasis.

In this study, probiotic strains that could or could not decrease IL-17 levels in other animal pathological models, including mice with asthma [29], constipation, and rheumatoid arthritis (as measured in other experiments, some data are not shown), were applied to IMQ-induced psoriasis-like mice. The purpose of this study was to (1) compare the ameliorating effects of different probiotic strains on the pathological characteristics of psoriasis; (2) compare suppressing effects of different probiotic strains on inflammatory cytokine levels associated with psoriasis; (3) compare modulating effects of different probiotic strains on gut microbiota; (4) perform correlation analysis to explore how probiotics ameliorate psoriasis.

## 2. Materials and Methods

### 2.1. Bacterial Strains

The strains used in this study were kept in the Culture Collection of Food Microorganisms (CCFM) of Jiangnan University (Wuxi, Jiangsu, China).

*Lactobacillus* strains were cultured at 37 °C for 16 h under aerobic conditions with de Man, Rogosa, and Sharpe (MRS) broth (Sinopharm Chemical Reagent, Shanghai, China). *Bifidobacterium* strains were cultured at 37 °C for 30 h under anaerobic conditions with modified MRS broth containing 0.05% (*w*/*v*) L-cysteine-HCl (Sinopharm Chemical Reagent). Detailed information on the probiotics is shown in Table 1. The strains were cultured for three generations and centrifuged at 8000 rpm for 10 min (Eppendorf, Hamburg, Germany) to remove the supernatant. The strains were washed with phosphate buffered-saline (PBS, Sinopharm Chemical Reagent) three times and then resuspended in normal saline at a concentration of 5 × 10^9^ colony-forming units (CFU)/mL.

### 2.2. Animal Experiments

Female BALB/c mice (6–8 weeks, 18–20 g, Beijing Vital River Laboratory Animal Technology, Beijing, China) were housed in a barrier condition. The temperature was 23 °C and the humidity was 55%. All animal procedures were performed according to the European Community guidelines (Directive 2010/63/EU) and approved by the Ethics Committee of Jiangnan University (JN. No20200515b1040630(066)).

**Figure 1:**
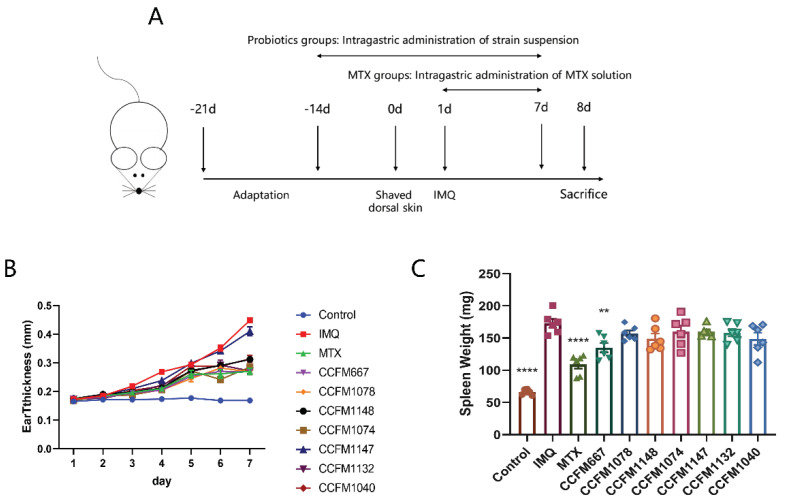
Effects of probiotics on psoriasis-like pathological characteristics. (**A**) Animal experiment design. (**B**) Ear thickness of mice. (**C**) Spleen weight of mice at D7. ** *p* < 0.01 and **** *p* < 0.0001.

After 1 week of adaptation, mice were separated into 10 groups: control group, IMQ (Aldara, 3M Pharmaceuticals, St. Paul, MN, USA) group, methotrexate (MTX, SPH Sine Pharmaceutical Laboratories, Shanghai, China) positive control group, and probiotic groups (seven groups). Six mice were included in each group. The probiotic groups were intra-gastrically administered 200 μL of strain suspension daily for 2 weeks, while the other mice received intragastric administration of the same volume of sterile saline. Two weeks later, shaving the dorsal skin and applying 62.5 mg IMQ cream to the dorsal skin and 20 mg to the right ear daily for seven consecutive days was performed [30], while mice in the control group received the same volume of Vaseline (Lircon Medical Technology, Dezhou, Shandong, China). During that time, probiotic groups received strain suspensions, the control and IMQ groups received sterile saline, while the MTX group received methotrexate dissolved in normal saline at 1 mg/kg/day [31,32]. Mice were sacrificed on the 8th day (Figure 1A).

### 2.3. Ear Thickness Determination and Dorsal Skin Score

The ear thickness of the mice was measured with a digital vernier calliper (Guanglu Measuring Instrument, Guilin, Guangxi, China) daily during the IMQ receiving period. The lesion skin of mice was scored in terms of the clinical psoriasis area and severity index (PASI). Severity of thickening, scaling, and erythema were scored separately on a scale from 0–4: 0, none; 1, slight; 2, moderate; 3, marked; 4, very marked. Cumulative Score was the sum of the scores of the three.

### 2.4. Skin Histopathology

After mice were sacrificed, the skin was removed, sliced, embedded in paraffin wax (Sigma Aldrich, St. Louis, MO, USA), and stained with haematoxylin and eosin (Sigma Aldrich) for further microscopic examination. The digital scanner (Pannoramic MIDI, 3DHistech, Budapest, Hungary) was used to scan photomicrographs (20×).

### 2.5. Skin Cytokine Analysis

Crushing dorsal skin samples (100 mg) in 900 μL radioimmunoprecipitation assay lysis buffer (Beyotime Biotechnology, Shanghai, China) containing 2% (*v*/*v*) protease inhibitor mixture (Beyotime Biotechnology) and 2% (*v*/*v*) phosphatase inhibitor mixture (Beyotime Biotechnology) with the grinder (Scientz48, Scientz Biotechnology, Ningbo, Zhejiang, China) at 60 Hz for 30 s, five times. According to the manufacturer’s instructions, IL-22, IL-23, and IL-17 levels were measured using enzyme-linked immunosorbent assay (ELISA) kits (Enzyme-linked Biotechnology, Shanghai, China), and total protein levels were measured using an enhanced bicinchoninic acid protein assay kit (Beyotime Biotechnology).

### 2.6. SCFA Analysis

SCFA levels in the caecum (50 mg) were measured by gas chromatography-mass spectrometer (GC-MS, Shimadzu Corp., Kyoto, Japan) using a previously published method [33].

### 2.7. 16S rRNA Amplification Sequencing of Faecal Samples

A swab (Huangchenyang Technology, Shenzhen, Guangzhou, China) was used to collect skin bacteria. Faecal samples and swab samples were collected the day before euthanasia and frozen at −80 °C. According to the manufacturer’s instructions, DNA from faecal samples was extracted using a Fast DNA spin kit for faeces (MP Biomedicals, Santa Ana, CA, USA). The extracted DNA was then amplified by polymerase chain reaction (PCR). The V4 region of faecal samples of the 16S rRNA gene were amplified using the primers 341F and 806R. The detailed protocol has been described previously [34]. PCR products were purified using a TIANgel mini purification kit (Tiangen Biotech, Beijing, China) and then sequenced using the Illumina sequencing platform (Miseq, Illumina, Santiago Canyon, CA, USA).

### 2.8. Bioinformatics Analysis

All sequences were analysed using Quantitative Insights into Microbial Ecology (QIIME2). Alpha and beta diversity analyses were also performed using QIIME2. Heatmap analysis was performed using the MetaboAnalyst website (https://www.metaboanalyst.ca/MetaboAnalyst/ModuleView.xhtml, accessed on November 2020). The data were normalised by the median, transformed by log transformation, and scaled by the centred mean. Heat tree analysis and linear discriminant analysis (LDA) effect size were performed using the MicrobiomeAnalyst website (https://www.microbiomeanalyst.ca/MicrobiomeAnalyst/upload/OtuUploadView.xhtml, accessed on December 2020). The low count filter was a 20% prevalence filter, whereas the low-variance filter was based on the inter-quantile range. The data were normalised using total sum scaling. Differences were regarded significant at *p* < 0.05. 

### 2.9. Statistical Analysis

Major statistical analyses were performed using GraphPad Prism (version 8, GraphPad, La Jolla, CA, USA) and the Statistical Package for the Social Sciences (version 22, SPSS, Chicago, IL, USA). Data are the mean ± standard error of the mean. The differences between multiple groups were calculated using one-way analysis of variance and Duncan’s multiple range test while *p* < 0.05 was considered to indicate statistical significance. * *p* < 0.05, ** *p* < 0.01, *** *p* < 0.001 and **** *p* < 0.0001. Differences between multiple groups were calculated using the Tukey–Kramer method, and differences were considered significant at *p* < 0.05.

## 3. Results

### 3.1. CCFM667, CCFM1078, CCFM1074, and CCFM1132 Ameliorated Psoriasis-Like Pathological Characteristics

In previous experiments, it was found that *B. adolescentis* CCFM667 reduced IL-17 levels in mice did ameliorate psoriasis, while *B. animalis* CCFM1148 and *L. paracasei* CCFM1147 failed to reduce IL-17 levels and did not. Therefore, in the selection of strains in this experiment, we selected strains that could or could not reduce the IL-17 level in other animal pathological models, including mice with asthma [29], constipation, and rheumatoid arthritis (as measured in other experiments, some data are not shown).

**Figure 2:**
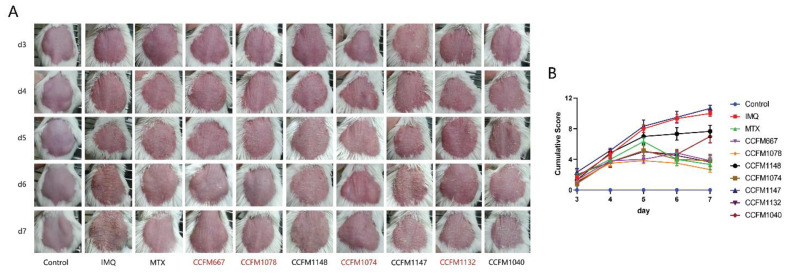
Effects of probiotics on dorsal skin. (**A**) Representative photographs of dorsal skin of mice from D3 to D7. (**B**) PASI of mice in different groups. Cumulative Score is the sum of the scores of the severity of thickening, scaling, and erythema.

Continuous application of IMQ resulted in thickening of the ears (Figure 1B), swelling of spleens (Figure 1C), and erythema, scaling, and thickening of the dorsal skin of mice (Figure 2). These results are consistent with literature reports [30,35,36]. Except for *L. paracasei* CCFM1147, all probiotics effectively ameliorated the swelling of ears. In the spleen, only MTX and *B. adolescentis* CCFM667 effectively ameliorated spleen swelling.

**Figure 3:**
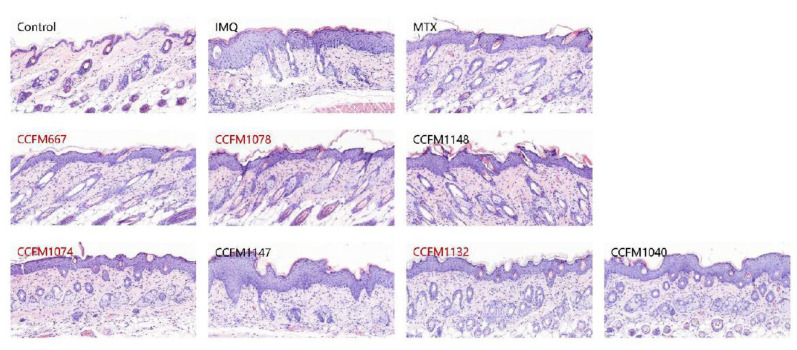
Representative photomicrographs of haematoxylin and eosin sections of dorsal skin tissue.

With respect to the pathological characteristics of dorsal skin, MTX, *B. adolescentis* CCFM667, *B. breve* CCFM1078, *L. paracasei* CCFM1074, and *L. reuteri* CCFM1132 effectively ameliorated erythema, scaling, and thickening, while *B. animalis* CCFM1148, *L. paracasei* CCFM1147, and *L. reuteri* CCFM1040 had almost no effect. Although the skin thickening in the *B. animalis* CCFM1148 group was not serious, this group had very serious erythema. Pathological sections showed that the epidermal structure of the control group consisted of only one or two layers of cells, while the epidermal structure of the IMQ group was clearly thickened (Figure 3). Consistent with the pathological characteristics, MTX, *B. adolescentis* CCFM667, *B. breve* CCFM1078, *L. paracasei* CCFM1074, and *L. reuteri* CCFM1132 effectively reduced epidermal thickness. Therefore, *B. adolescentis* CCFM667, *B. breve* CCFM1078, *L. paracasei* CCFM1074, and *L. reuteri* CCFM1132 were considered effective in relieving psoriasis-like pathological characteristics, while *B. animalis* CCFM1148, *L. paracasei* CCFM1147, and *L. reuteri* CCFM1040 were considered to be ineffective strains.

### 3.2. CCFM667, CCFM1078, CCFM1074, and CCFM1132 Suppressed the Psoriasis-Like Immune Response

**Figure 4:**
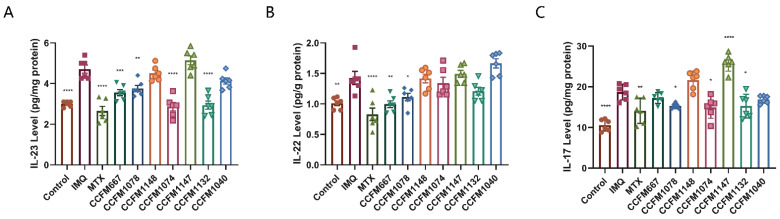
Effects of probiotics on levels of IL-23/Th17 axis-associated inflammatory cytokines. (**A**) IL-23 levels (*n* = 6). (**B**) IL-22 levels (*n* = 6). (**C**) IL-17 levels (*n* = 6). * *p* < 0.05, ** *p* < 0.01, *** *p* < 0.001 and **** *p* < 0.0001.

Psoriasis is an immune-mediated skin disease, and the IL-23/Th17 axis takes part in its progression [5,6]. IL-23 drives the differentiation of Th17 cells, and Th17 cells produce IL-22 and IL-17 [10]. Therefore, levels of IL-23, IL-22, and IL-17 in skin lesions of mice were determined. Continuous application of IMQ resulted in increased levels of IL-23, IL-22, and IL-17, while MTX significantly suppressed this trend (Figure 4). In the probiotic groups, *B. breve* CCFM1078 significantly suppressed expression of all three cytokines, while *B. adolescentis* CCFM667, *L. paracasei* CCFM1074, and *L. reuteri* CCFM1132 significantly suppressed expression of two cytokines. *B. animalis* CCFM1148, *L. paracasei* CCFM1147, and *L. reuteri* CCFM1040 had no effect on ameliorating psoriasis-like symptoms and did not suppress the psoriasis-like immune response (Figure 2). Therefore, it was concluded that *B. adolescentis* CCFM667, *B. breve* CCFM1078, *L. paracasei* CCFM1074, and *L. reuteri* CCFM1132 were effective in ameliorating psoriasis through the IL-23/Th17 axis.

### 3.3. Probiotics Exerted Different Effects on SCFA Metabolism

**Figure 5:**
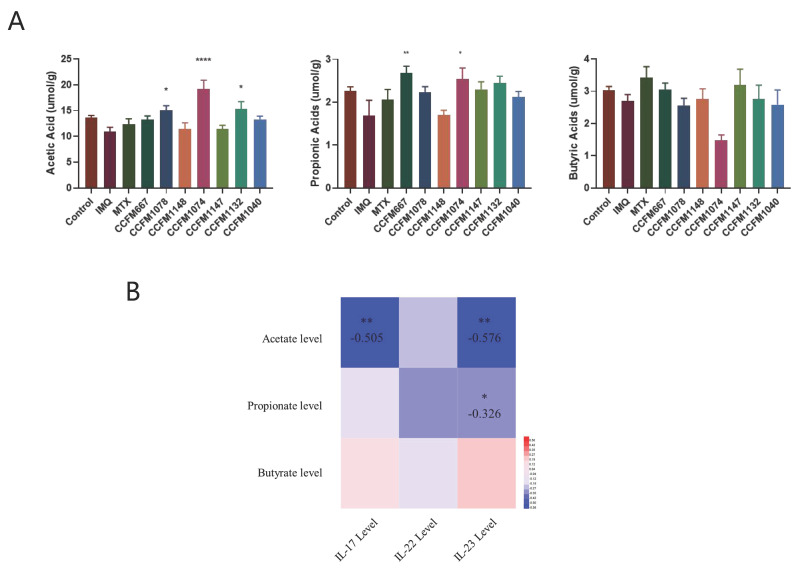
Effects of probiotics on short chain fatty acid metabolism. (**A**) Acetate, propionate, and butyrate levels in different groups (*n* = 6). (**B**) Spearman correlation analysis between the levels of SCFAs and the levels of IL-23/Th17 axis-associated inflammatory cytokines. * *p* < 0.05, ** *p* < 0.01 and **** *p* < 0.0001.

The main products of intestinal microbial fermentation are SCFAs, especially acetate, propionate, and butyrate [37]. Acetate, propionate, and butyrate levels were not significantly different in the control and IMQ groups (Figure 5A). Compared to the IMQ groups, acetate level in the *B. breve* CCFM1078, *L. paracasei* CCFM1074, and *L. reuteri* CCFM1132 groups significantly increased, while propionate levels in the *B. adolescentis* CCFM667 and *L. paracasei* CCFM1074 groups significantly increased, but butyrate level in all groups showed no significant difference. In short, the strains that effectively ameliorated psoriasis increased the levels of acetate or propionate.

Further correlation analysis was performed between the levels of SCFAs in the colon content and the levels of inflammatory cytokines in the skin tissue; the result showed that the level of acetate was significantly negatively correlated with the levels of IL-17 and IL-23, and the level of propionate was significantly negatively correlated with the levels of IL-23 (Figure 5B). It was concluded that probiotics that effectively alleviated psoriasis could inhibit the release of IL-23/Th17 axis inflammatory cytokines by promoting the production of SCFAs.

### 3.4. Probiotics Exerted Different Effects on Gut Microbial Composition

**Figure 6:**
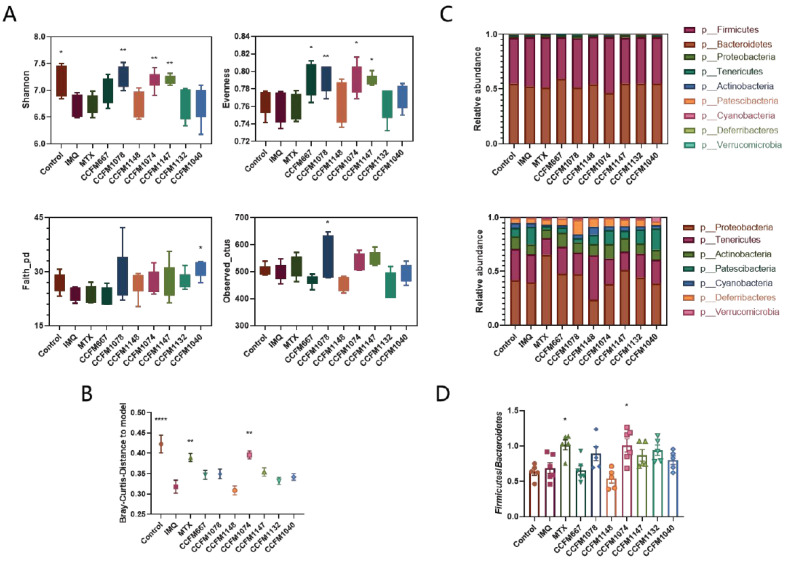
Effects of probiotics on gut microbial composition. (**A**) Alpha diversity and (**B**) beta diversity of gut microbiota (*n* = 5/6). (**C**) The relative abundance of gut microbiota at the phylum level (*n* = 5/6). OUT, operational taxonomic unit. (**D**) The rate of Firmicutes to Bacteroidetes. * *p* < 0.05, ** *p* < 0.01.

Changes in gut microbial diversity in patients with psoriasis have been reported in a large number of studies [18,19,21]. The α diversity is shown in Figure 6A, based on different indicators, including the Shannon, Evenness, and Faith indices, and observed operational taxonomic units. Considering species and abundance, the Shannon index showed that compared to the control group, the α diversity of the IMQ group significantly decreased, while application of *B. breve* CCFM1078, *L. paracasei* CCFM1074, and *L. paracasei* CCFM1147 significantly suppressed this trend. However, this trend was not observed when the evolutionary relationship was taken into consideration, as shown by the Faith index. The β diversity is shown in Figure 6B, based on the Bray–Curtis distance, which reveals the decline of common species between the control and IMQ groups. Only MTX and *L. paracasei* CCFM1074, which both relieved psoriasis-like pathological characteristics, suppressed this trend. The relative abundance at the phylum level is shown in Figure 6C. Bacteroidetes and Firmicutes had the highest abundance, and the ratio of Firmicutes to Bacteroidetes (F/B) in the control and IMQ groups was not significantly different, while MTX and *L. paracasei* CCFM1074, which both relieved psoriasis-like pathological characteristics, increased the F/B ratio (Figure 6D). These results showed that probiotics exerted different effects on the regulation of gut microbial composition.

### 3.5. Key Differences between Effective and Ineffective Probiotics in Ameliorating Psoriasis

**Figure 7:**
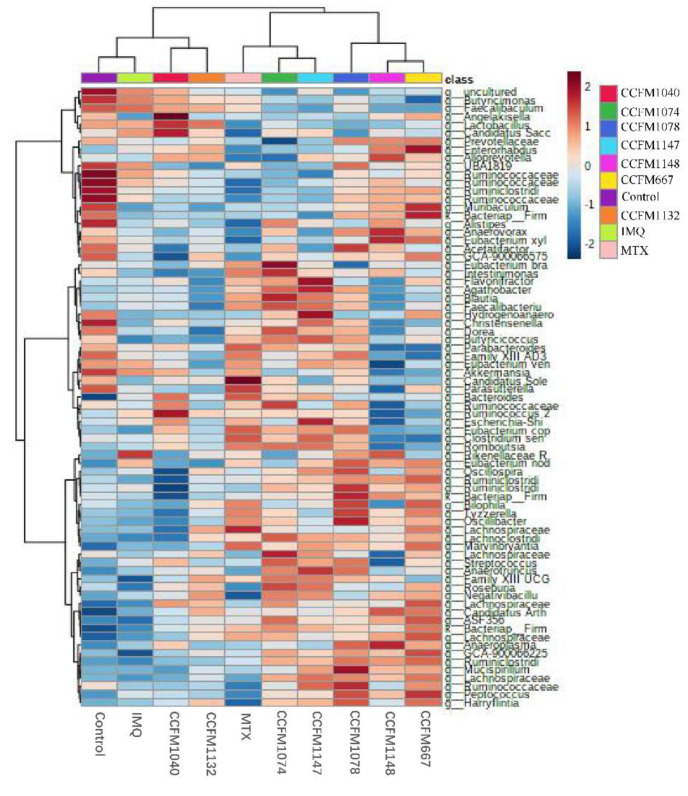
Clustering analysis of gut microbiota at the genus level (*n* = 5/6).

To better explore the effect of probiotics on gut microbial composition, the gut microbiota at the genus level in different groups of mice was analysed using heatmap analysis (Figure 7). Although the control and IMQ groups were clustered into the same category, the abundance of some gut microbiota in the IMQ group was significantly reduced. In addition, *L. reuteri* CCFM1040 and CCFM1132, *L. paracasei* CCFM1074 and CCFM1137, and *Bifidobacterium* CCFM1078, CCFM1148, and CCFM667 were clustered into the same category. Therefore, it was speculated that the effects of probiotics of the same genus or species were similar in gut microbial composition in general, but there should be some key differences that made certain probiotics effective in ameliorating psoriasis, while others were ineffective.

**Figure 8:**
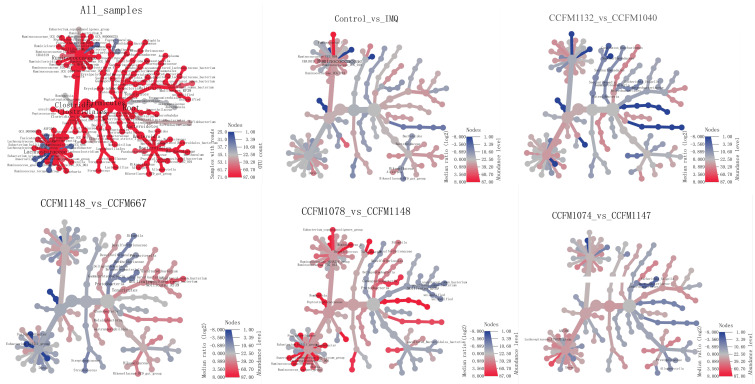
Heat tree analysis of gut microbiota at the genus level (*n* = 5/6). Nodes marked with text represent a significance difference between the two groups, *p* < 0.05.

To explore the key differences that made certain probiotics effective in ameliorating psoriasis, heat tree analysis [38] was performed. Differential gut microbiota between the control and IMQ groups, as well as *L. reuteri* CCFM1040 and CCFM1132 groups, *L. paracasei* CCFM1074, and CCFM1137 groups, *Bifidobacterium* CCFM1148, and CCFM667 groups, and *Bifidobacterium* CCFM1148, and CCFM1078 groups are shown in Figure 8 (*p* < 0.05).

**Figure 9:**
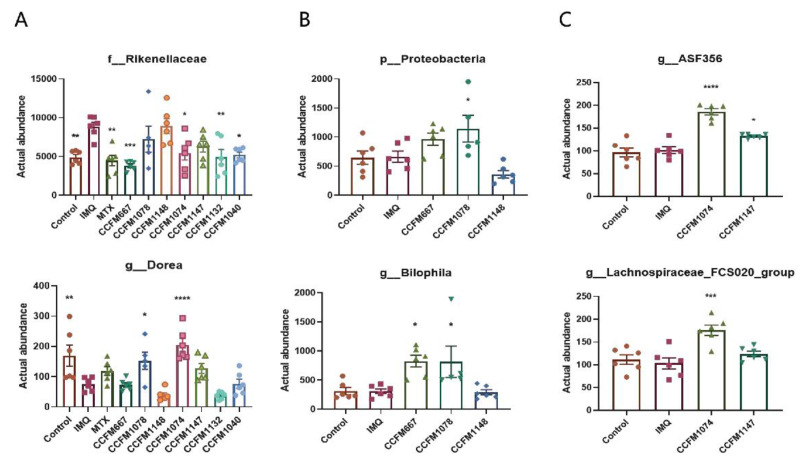
Difference analysis of gut microbiota between groups (*n* = 5/6). The abundance of key differential gut microbiota between (**A**) the control and IMQ groups, (**B**) the *Bifidobacterium* CCFM1078, CCFM667, CCFM1148 groups, (**C**) the *L. paracasei* CCFM1074 and CCFM1147 groups. * *p* < 0.05, ** *p* < 0.01, *** *p* < 0.001 and **** *p* < 0.0001.

Based on heat tree analysis, the abundance of significantly differentiated gut microbiota was analysed further (Figure 9). Figure 9A shows the changes in the abundance of the family Rikenellaceae and that the genus *Dorea* in the IMQ group was suppressed by the effective strains, while the ineffective strains did not have such function. Figure 9B shows the increase in the abundance of the phylum Proteobacteria and the genus *Bilophila* in the effective *Bifidobacterium* CCFM1078 and CCFM667 groups compared to the ineffective *Bifidobacterium* CCFM1148 group. Figure 9C shows the increase in the abundance of the species *ASF356* and *Lachnospiraceae FCS020* group in the effective *L. paracasei* CCFM1074 group compared to the ineffective *L. paracasei* CCFM1147 group. This phenomenon was not found in the *L. reuteri* CCFM1040 and CCFM1132 groups. It was suggested that the above changes in the abundance of gut microbiota may be responsible for the different effects of the strains on psoriasis.

**Figure 10:**
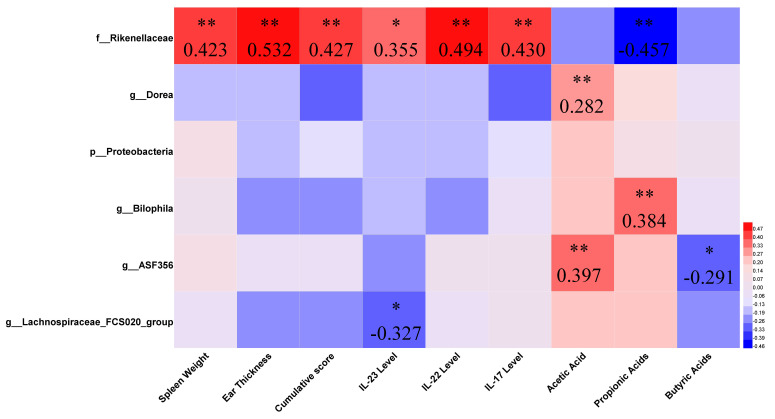
Spearman correlation analysis between the key gut microbiota and psoriasis-like pathological characteristics. * *p* < 0.05, ** *p* < 0.01.

To further explore the role of the above differential gut microbiota, a correlation analysis was performed (Figure 10). The family Rikenellaceae, which was different between the control and IMQ groups, was positively correlated with spleen weight, ear thickness, cumulative scores, and IL-23/Th17 axis-associated inflammatory factor levels, while it was negatively correlated with the propionate level. This suggested that the development of psoriasis may lead to an increase in the abundance of the family Rikenellaceae, while *B. adolescentis* CCFM667, *L. paracasei* CCFM1074, and *L. reuteri* CCFM1132 reduced its abundance (Figure 9A). The *Lachnospiraceae FCS020* group, which was different between the CCFM1074 and CCFM1147 groups (Figure 9C), was negatively correlated with IL-23 level. This suggested that the development of psoriasis may lead to a decrease in the abundance of the *Lachnospiraceae FCS020* group, while CCFM1074 promoted its abundance. In addition, the genera *Dorea* and *Bilophila*, and the species *ASF356* were significantly correlated with SCFA levels, but they did not correlate with psoriasis-like pathological characteristics.

In summary, the increase in the abundance of the family Rikenellaceae may be related to the development of psoriasis, while *B. adolescentis* CCFM667, *L. paracasei* CCFM1074, and *L. reuteri* CCFM1132 had the effect of restraining this trend.

## 4. Discussion

Psoriasis is an immune-mediated skin disease, and the IL-23/Th17 axis plays an important part in its progression. In the IL-23/Th17 axis, IL-23 induces the differentiation of Th17 cells and then promotes the secretion of IL-22 and IL-17, which eventually leads to the aggravation of psoriasis [39]. IL-23 inhibitors, such as guselkumab, risankizumab and tildrakizumab, are used in the management of psoriasis and other Th17 mediated inflammatory diseases [40,41]. Besides this, IL-17 inhibitors, including secukinumab, ixekizumab, and brodalumab [11], are also widely used to relieve psoriasis. Probiotics can affect the differentiation of T-lymphocyte through their metabolites on T-lymphocytes, thereby affecting the number of Th17 cells in the host, and thus affecting IL-17 levels. A study has reported that obese people have lower levels of IL-17 in their blood after ingesting yogurt containing probiotics [42]. Based on this, probiotic strains that could or could not decrease IL-17 levels in other animal pathological models, including mice with asthma [29], constipation, and rheumatoid arthritis (as measured in other experiments, some data are not shown) were selected and applied to IMQ-induced psoriasis-like mice to compare the ameliorating effects of the strain on psoriasis. ELISA results showed that *B. adolescentis* CCFM667, *B. breve* CCFM1078, *L. paracasei* CCFM1074, and *L. reuteri* CCFM1132 decreased levels of IL-23/Th17 axis-related inflammatory cytokines, including IL-23, IL-22, and IL-17, while *B. animalis* CCFM1148, *L. paracasei* CCFM1147, and *L. reuteri* CCFM1040 did not. Similarly, *B. adolescentis* CCFM667, *B. breve* CCFM1078, *L. paracasei* CCFM1074, and *L. reuteri* CCFM1132 ameliorated psoriasis-like pathological characteristics, including skin lesion PASI scores, ear thickness, and spleen weight, while *B. animalis* CCFM1148, *L. paracasei* CCFM1147, and *L. reuteri* CCFM1040 did not. Based on the above results, it is preliminarily believed that probiotics have the potential to ameliorate psoriasis by decreasing the levels of IL-23/Th17 axis-related inflammatory cytokines. This is consistent with that reported by Zuzana et al., who declared that gut microbiota directly regulates imiquimod-induced skin inflammation [43]. Other studies have also reported the function of probiotics in decreasing levels of IL-23/Th17 axis-related inflammatory cytokines [44,45].

In recent years, increasing attention has been paid to the relationship between intestinal microecology and human health. SCFAs, especially acetate, propionate, and butyrate [37], are the main products of gut microbial fermentation [37]. It has been reported that SCFAs could promote the differentiation of Treg cells and then affect the balance of Th17/Treg, so as to alleviate the occurrence of psoriasis [17,46]. Furthermore, Khyshiktuev et al. have found decreased levels of acetate and propionate in the serum of patients with psoriasis [47]. In this study, *B. breve* CCFM1078, *L. paracasei* CCFM1074, and *L. reuteri* CCFM1132 increased acetate levels, and *B. adolescentis* CCFM667 and *L. paracasei* CCFM1074 increased propionate levels, while the ineffective strains *B. animalis* CCFM1148, *L. paracasei* CCFM1147, and *L. reuteri* CCFM1040 did neither. This suggested that acetate and propionate played a role in the alleviation of psoriasis. Besides this, the levels of acetate and propionate were significantly negatively correlated with the levels of IL-23/Th17 axis-related inflammatory cytokines. It was concluded that probiotics that effectively alleviated psoriasis could inhibit the release of IL-23/Th17 axis inflammatory cytokines by promoting the production of SCFAs.

Human health is closely related to the changes of gut microbiota, and a large number of studies have reported the changes of intestinal flora diversity in patients with psoriasis [19,20,48]. Many studies have also reported that there is a significant decrease in gut microbial diversity [17,18]. In this study, the α and β diversity of the gut microbiota in psoriasis-like mice decreased, and the relative abundance changed. Some studies report an increased ratio of F/B in patients with psoriasis, which is associated with altered carbohydrate metabolism of medium-chain fatty acids and SCFAs [19,49], while Masallat et al. have found that the F/B ratio is positively correlated with the PASI score [20]. However, in this study, the F/B ratio in the control and IMQ groups showed no significant difference, but was increased in the MTX and CCFM1074 groups, which both relieved psoriasis-like pathological characteristics. However, Scher et al. report a decreased abundance of Bacteroidetes in patients with psoriasis [18]. Therefore, the role of F/B ratio in the development of psoriasis needs further study.

Some studies have reported strain-specific effects of probiotics in ameliorating disease [50,51]. In this study, heatmap analysis showed that probiotics of the same genus or species had similar effects in modulating gut microbiota, although the effects of these probiotics in ameliorating psoriasis were different. Based on this, the abundance of gut microbiota was compared between the effective and ineffective probiotic groups, and some key differential gut microbiota were identified. The abundance of the family Rikenellaceae in the IMQ group significantly increased, while the effective strains, *B. adolescentis* CCFM667, *L. paracasei* CCFM1074, and *L. reuteri* CCFM1132, decreased its abundance. Meanwhile, the abundance of the family Rikenellaceae was positively correlated with psoriasis-like pathological characteristics and negatively correlated with propionate level. This suggested that the development of psoriasis may have correlation with the increase in the abundance of the family Rikenellaceae. Similarly, Sun et al. have found that the Th1 response in the colon of mice is enhanced after receiving antibiotics, while the abundance of the family Rikenellaceae increased [52], suggesting that Rikenellaceae abundance is related to immunity, but its specific mechanism remains unknown. Compared to the *L. paracasei* CCFM1147 group, *L. paracasei* CCFM1074 increased the abundance of *the Lachnospiraceae FCS020 group*, which was positively correlated with IL-23 levels. This suggested that the development of psoriasis may lead to a decrease in the abundance of *the Lachnospiraceae FCS020 group*, which was related to inflammatory responses. The key differential gut microbiota in the *B. breve* CCFM1078 group was not identified, but the *B. breve* CCFM1078 strain largely ameliorated psoriasis, and was most successful in suppressing inflammatory responses of all the probiotic groups; therefore, it was speculated that *B. breve* CCFM1078 ameliorated psoriasis directly through the IL-23/IL-17 axis rather than exerting effects on gut microbiota. Due to the strain-specificity, this study believes that the effective strains can be developed into products to enable patients to increase the intake of the effective strains, thereby ameliorating psoriasis. In addition, probiotics could only alleviate psoriasis to a certain extent, and could not replace drugs. Probiotic intake should be used as a supplement to medication

In this study, we found that probiotics could ameliorate the pathological characteristics of psoriasis in mice by inhibiting the IL-23/Th17 axis-related inflammatory response and regulating gut microbiota, providing a new possibility for the treatment of psoriasis. However, there are still shortcomings in this study, such as the lack of repeated experiments to verify the results. In addition, tests of immune cells were lacking to strengthen the link between gut microbial composition and immunological changes in the skin. These will be the direction of future research. In the future, this study will further confirm whether the effective strains have the same phenomenon and effects in the human body, and further explore the mechanisms.

## 5. Conclusions

In summary, the effects of seven probiotic strains in ameliorating psoriasis were explored in IMQ-induced psoriasis-like mice. *B. adolescentis* CCFM667, *B. breve* CCFM1078, *L. paracasei* CCFM1074, and *L. reuteri* CCFM1132 ameliorated psoriasis via suppressing the release of IL-23/Th17 axis-related inflammatory factors by promoting the content of SCFAs, while *B. animalis* CCFM1148, *L. paracasei* CCFM1147, and *L. reuteri* CCFM1040 did not have such effects. Among these, *B. breve* CCFM1078 exhibited the best anti-inflammatory effects. The development of psoriasis may lead to an increase in the abundance of the family Rikenellaceae while the effective strains *B. adolescentis* CCFM667, *L. paracasei* CCFM1074, and *L. reuteri* CCFM1132 decreased its abundance. These results revealed differences in the regulation of immune response and gut microbiota by strains, so it was confirmed that effects were strain-specificity. This study demonstrated the potential of probiotics in the treatment of psoriasis.

## Figures and Tables

**Table 1:** Information of strains.

Strain Number	Strain Original Number	Genus/Species	Origin
CCFM667	CCFM667	*Bifidobacterium adolescentis*	CCFM
CCFM1078	JSWX17M1	*Bifidobacterium breve*	
CCFM1148	JSWX23M8	*Bifidobacterium animalis*	
CCFM1074	FJSWX1M3	*Lacticaseibacillus paracasei*	
CCFM1147	VCQQJ4174M3		
CCFM1032	FZJTZ20M3	*Limosilactobacillus reuteri*	
CCFM1040	FYNDL13		

CCFM: The Culture Collection of Food Microorganisms of Jiangnan University (Wuxi, Jiangsu, China).

## Data Availability

Data sharing not applicable.

## Abbreviations

CCFM	Culture Collection of Food Microorganisms
EILISA	Enzyme-linked Immunosorbent Assay
IL	Interleukin
IMQ	Imiquimod
LDA	Linear Discriminant Analysis
MRS	Man, Rogosa and Sharpe
MTX	Methotrexate
OD	Optical density
PASI	Psoriasis Area and Severity Index
PCA	Principal Components Analysis
PCR	Polymerase Chain Reaction
SCFAs	Short-Chain Fatty Acids
Th	Helper T cells
Tregs	Regulatory cells

## References

[1] Grayson M. (2012). Psoriasis. Nature.

[2] Christophers E. (2001). Psoriasis–Epidemiology and clinical spectrum. Clin. Exp. Derm..

[3] Parisi R., Symmons D.P., Griffiths C.E., Ashcroft D.M. (2013). Global epidemiology of psoriasis: A systematic review of incidence and prevalence. J. Investig. Derm..

[4] Ferreli C., Pinna A.L., Pilloni L., Tomasini C.F., Rongioletti F. (2018). Histopathological aspects of psoriasis and its uncommon variants. G. Ital. Derm. Venereol..

[5] Griffiths C.E., Barker J.N. (2007). Pathogenesis and clinical features of psoriasis. Lancet.

[6] Greb J.E., Goldminz A.M., Elder J.T., Lebwohl M.G., Gladman D.D., Wu J.J., Mehta N.N., Finlay A.Y., Gottlieb A.B. (2016). Psoriasis. Nat. Rev. Dis. Primers.

[7] Schn M.P. (2019). Adaptive and innate immunity in psoriasis and other inflammatory disorders. Front. Immunol..

[8] Diani M., Altomare G., Reali E. (2016). T helper cell subsets in clinical manifestations of psoriasis. J. Immunol. Res..

[9] Paust H.J., Turner J.E., Steinmetz O.M., Peters A., Heymann F., Holscher C., Wolf G., Kurts C., Mittrucker H.W., Stahl R.A. (2009). The IL-23/Th17 axis contributes to renal injury in experimental glomerulonephritis. J. Am. Soc. Nephrol..

[10] Boehncke W.H., Schon M.P. (2015). Psoriasis. Lancet.

[11] Kamata M., Tada Y. (2018). Safety of biologics in psoriasis. J. Derm..

[12] Thursby E., Juge N. (2017). Introduction to the human gut microbiota. Biochem. J..

[13] He S., Ivanova N., Kirton E., Allgaier M., Bergin C., Scheffrahn R.H., Kyrpides N.C., Warnecke F., Tringe S.G., Hugenholtz P. (2013). Comparative metagenomic and metatranscriptomic analysis of hindgut paunch microbiota in wood- and dung-feeding higher termites. PLoS ONE.

[14] Dominguez-Bello M.G., Godoy-Vitorino F., Knight R., Blaser M.J. (2019). Role of the microbiome in human development. Gut.

[15] Tyagi A.M., Yu M., Darby T.M., Vaccaro C., Li J.Y., Owens J.A., Hsu E., Adams J., Weitzmann M.N., Jones R.M. (2018). The microbial metabolite butyrate stimulates bone formation via T regulatory cell-mediated regulation of WNT10B expression. Immunity.

[16] Chen L., Sun M., Wu W., Yang W., Huang X., Xiao Y., Ma C., Xu L., Yao S., Liu Z. (2019). Microbiota metabolite butyrate differentially regulates Th1 and Th17 cells’ differentiation and function in induction of colitis. Inflamm. Bowel Dis..

[17] Hidalgo-Cantabrana C., Gomez J., Delgado S., Requena-Lopez S., Queiro-Silva R., Margolles A., Coto E., Sanchez B., Coto-Segura P. (2019). Gut microbiota dysbiosis in a cohort of patients with psoriasis. Br. J. Derm..

[18] Scher J.U., Ubeda C., Artacho A., Attur M., Isaac S., Reddy S.M., Marmon S., Neimann A., Brusca S., Patel T. (2015). Decreased bacterial diversity characterizes the altered gut microbiota in patients with psoriatic arthritis, resembling dysbiosis in inflammatory bowel disease. Arthritis Rheumatol..

[19] Chen Y.J., Ho H.J., Tseng C.H., Lai Z.L., Shieh J.J., Wu C.Y. (2018). Intestinal microbiota profiling and predicted metabolic dysregulation in psoriasis patients. Exp. Derm..

[20] Doaa M., Dalia M., Ahmed F.S. (2016). Gut bacterial microbiota in psoriasis: A case control study. Afr. J. Microbiol. Res..

[21] Codoner F.M., Ramirez-Bosca A., Climent E., Carrion-Gutierrez M., Guerrero M., Perez-Orquin J.M., Horga de la Parte J., Genoves S., Ramon D., Navarro-Lopez V. (2018). Gut microbial composition in patients with psoriasis. Sci. Rep..

[22] Tan L., Zhao S., Zhu W., Wu L., Li J., Shen M., Lei L., Chen X., Peng C. (2018). The Akkermansia muciniphila is a gut microbiota signature in psoriasis. Exp. Derm..

[23] Wolters M. (2005). Diet and psoriasis: Experimental data and clinical evidence. Br. J. Derm..

[24] Barrea L., Megna M., Cacciapuoti S., Frias-Toral E., Fabbrocini G., Savastano S., Colao A., Muscogiuri G. (2020). Very low-calorie ketogenic diet (VLCKD) in patients with psoriasis and obesity: an update for dermatologists and nutritionists. Crit. Rev. Food Sci..

[25] Cosme-Silva L., Dal-Fabbro R., Cintra L.T.A., Ervolino E., Plazza F., Mogami Bomfim S., Duarte P.C.T., Junior V., Gomes-Filho J.E. (2020). Reduced bone resorption and inflammation in apical periodontitis evoked by dietary supplementation with probiotics in rats. Int. Endod. J..

[26] Li X., Hu D., Tian Y., Song Y., Hou Y., Sun L., Zhang Y., Man C., Zhang W., Jiang Y. (2020). Protective effects of a novel Lactobacillus rhamnosus strain with probiotic characteristics against lipopolysaccharide-induced intestinal inflammation in vitro and in vivo. Food Funct..

[27] Chen Y.H., Wu C.S., Chao Y.H., Lin C.C., Tsai H.Y., Li Y.R., Chen Y.Z., Tsai W.H., Chen Y.K. (2017). Lactobacillus pentosus GMNL-77 inhibits skin lesions in imiquimod-induced psoriasis-like mice. J. Food Drug Anal..

[28] Rather I.A., Bajpai V.K., Huh Y.S., Han Y.K., Bhat E.A., Lim J., Paek W.K., Park Y.H. (2018). Probiotic Lactobacillus sakei proBio-65 extract ameliorates the severity of imiquimod induced psoriasis-like skin inflammation in a mouse model. Front. Microbiol..

[29] Li L., Fang Z., Lee Y.K., Zhao J., Zhang H., Lu W., Chen W. (2020). Prophylactic effects of oral administration of Lactobacillus casei on house dust mite-induced asthma in mice. Food Funct..

[30] van der Fits L., Mourits S., Voerman J.S., Kant M., Boon L., Laman J.D., Cornelissen F., Mus A.M., Florencia E., Prens E.P. (2009). Imiquimod-induced psoriasis-like skin inflammation in mice is mediated via the IL-23/IL-17 axis. J. Immunol..

[31] Zhao J., Di T., Wang Y., Wang Y., Liu X., Liang D., Li P. (2016). Paeoniflorin inhibits imiquimod-induced psoriasis in mice by regulating Th17 cell response and cytokine secretion. Eur. J. Pharm..

[32] Baker H., Ryan T.J. (1968). Methotrexate in psoriasis. Lancet.

[33] Mao B., Li D., Ai C., Zhao J., Zhang H., Chen W. (2016). Lactulose differently modulates the composition of luminal and mucosal microbiota in C57BL/6J mice. J. Agric. Food Chem..

[34] Tian P., Wang G., Zhao J., Zhang H., Chen W. (2019). Bifidobacterium with the role of 5-hydroxytryptophan synthesis regulation alleviates the symptom of depression and related microbiota dysbiosis. J. Nutr. Biochem..

[35] Ru Y., Li H., Zhang R., Luo Y., Song J., Kuai L., Xing M., Hong S., Sun X., Ding X. (2020). Role of keratinocytes and immune cells in the anti-inflammatory effects of Tripterygium wilfordii Hook. f. in a murine model of psoriasis. Phytomedicine.

[36] Zeng J., Lei L., Zeng Q., Yao Y., Wu Y., Li Q., Gao L., Du H., Xie Y., Huang J. (2020). Ozone therapy attenuates NF-kappaB-mediated local inflammatory response and activation of Th17 cells in treatment for psoriasis. Int. J. Biol. Sci..

[37] Koh A., De Vadder F., Kovatcheva-Datchary P., Backhed F. (2016). From dietary fiber to host physiology: Short-chain fatty acids as key bacterial metabolites. Cell.

[38] Foster Z.S., Sharpton T.J., Grunwald N.J. (2017). Metacoder: An R package for visualization and manipulation of community taxonomic diversity data. PLoS Comput. Biol..

[39] Di Cesare A., Di Meglio P., Nestle F.O. (2009). The IL-23/Th17 axis in the immunopathogenesis of psoriasis. J. Investig. Derm..

[40] Roberti R., Iannone L.F., Palleria C., De Sarro C., Spagnuolo R., Barbieri M.A., Vero A., Manti A., Pisana V., Fries W. (2020). Safety profiles of biologic agents for inflammatory bowel diseases: a prospective pharmacovigilance study in Southern Italy. Curr. Med. Res. Opin..

[41] Dattola A., Silvestri M., Tamburi F., Amoruso G.F., Bennardo L., Nistico S.P. (2020). Emerging role of anti-IL23 in the treatment of psoriasis: When humanized is very promising. Dermatol. Ther..

[42] Zarrati M., Salehi E., Mofid V., Hosseinzadeh-Attar M.J., Nourijelyani K., Bidad K., Shidfar F. (2013). Relationship between probiotic consumption and IL-10 and IL-17 secreted by PBMCs in overweight and obese people. Iran. J. Allergy Asthm..

[43] Stehlikova Z., Kostovcikova K., Kverka M., Rossmann P., Dvorak J., Novosadova I., Kostovcik M., Coufal S., Srutkova D., Prochazkova P. (2019). Crucial role of microbiota in experimental psoriasis revealed by a gnotobiotic mouse model. Front. Microbiol..

[44] Leccese G., Bibi A., Mazza S., Facciotti F., Caprioli F., Landini P., Paroni M. (2020). Probiotic Lactobacillus and Bifidobacterium strains counteract adherent-invasive Escherichia coli (AIEC) virulence and hamper IL-23/Th17 axis in ulcerative colitis, but not in crohn’s disease. Cells.

[45] Chen L., Zou Y., Peng J., Lu F., Yin Y., Li F., Yang J. (2015). Lactobacillus acidophilus suppresses colitis-associated activation of the IL-23/Th17 axis. J. Immunol. Res..

[46] Eppinga H., Sperna Weiland C.J., Thio H.B., van der Woude C.J., Nijsten T.E., Peppelenbosch M.P., Konstantinov S.R. (2016). Similar depletion of protective Faecalibacterium prausnitzii in psoriasis and inflammatory bowel disease, but not in hidradenitis suppurativa. J. Crohn’s. Colitis.

[47] Khyshiktuev B.S., Karavaeva T.M., Fal’ko E.V. (2008). Variability of quantitative changes in short-chain fatty acids in serum and epidermis in psoriasis. Klin. Lab. Diagn..

[48] Rinninella E., Raoul P., Cintoni M., Franceschi F., Miggiano G., Gasbarrini A., Mele M. (2019). What is the healthy gut microbiota composition? A changing ecosystem across age, environment, diet, and diseases. Microorganisms.

[49] Myers B., Brownstone N., Reddy V., Chan S., Thibodeaux Q., Truong A., Bhutani T., Chang H.W., Liao W. (2019). The gut microbiome in psoriasis and psoriatic arthritis. Best Pract. Res. Clin. Rheumatol..

[50] Fang Z., Li L., Liu X., Lu W., Zhao J. (2019). Strain-specific ameliorating effect of Bifidobacterium longum on atopic dermatitis in mice. J. Funct. Foods.

[51] Zhai R., Xue X., Zhang L., Yang X., Zhao L., Zhang C. (2019). Strain-specific anti-inflammatory properties of two Akkermansia muciniphila strains on chronic colitis in mice. Front. Cell Infect. Microbiol..

[52] Sun L., Zhang X., Zhang Y., Zheng K., Xiang Q., Chen N., Chen Z., Zhang N., Zhu J., He Q. (2019). Antibiotic-induced disruption of gut microbiota alters local metabolomes and immune responses. Front. Cell Infect. Microbiol..

